# Etanercept Attenuates Myocardial Ischemia/Reperfusion Injury by Decreasing Inflammation and Oxidative Stress

**DOI:** 10.1371/journal.pone.0108024

**Published:** 2014-09-26

**Authors:** Mei Yang, Jianchang Chen, Jing Zhao, Mei Meng

**Affiliations:** 1 Department of Critical Care Medicine, the Third Hospital of Jinan, Jinan, People’s Republic of China; 2 Department of Emergency, Shandong Provincial Hospital, Jinan, People’s Republic of China; 3 Department of Cardiology, Qilu Hospital of Shandong University, Jinan, People’s Republic of China; 4 Department of Critical Care Medicine, Shandong Provincial Hospital, Jinan, People’s Republic of China; Emory University, United States of America

## Abstract

The protective role of etanercept in myocardial ischemia/reperfusion is not well understood. The aim of this study was to investigate whether etanercept modulates neutrophil accumulation, TNF-α induction and oxidative stress in an ischemia/reperfusion injured rat heart model. Rats were randomly exposed to sham operation, myocardial ischemia/reperfusion (MI/R) alone, MI/R+ etanercept. The results demonstrated that compared to MI/R, etanercept reduced myocardial infarction area, myocardial myeloperoxidase (MPO) levels, serum creatinine kinase (CK) and lactate dehydrogenase (LDH) levels, and both serum and myocardial TNF-α production. Etanercept also markedly enhanced the activities of antioxidant enzymes superoxide dismutase (SOD) and glutathione peroxidase (GSH-PX), and reduced the level of malondialdehyde (MDA) in MI/R rats. In summary, our data suggested that etanercept has protective effects against MI/R injury in rats, which may be attributed to attenuating inflammation and oxidative stress.

## Introduction

The inflammatory reaction induced by ischemia/reperfusion is one of the most important links in the myocardial ischemia-reperfusion injury [1]. In the process of inflammation, various cytokines are released, including tumor necrosis factor α (TNF-α), interleukin 6 (IL-6) and IL-8, etc. [2]. TNF-α can trigger the inflammatory reaction caused by myocardial ischemia-reperfusion. In addition, vascular endothelial cell injury, and inflammatory cells, such as neutrophils, activated by cytokines and adhesion molecules are also included in inflammation. So TNF-α activity and the amount of neutrophil infiltration can be considered as the indicators of inflammatory reaction. Oxidative stress also plays a pivotal role in myocardial ischemia/reperfusion injury [3]. TNF-α plays a pivotal role in injury induced by various immune responses. Therefore, researches targeted at TNF-α draw much attention. Previous study has suggested that TNF inhibition after infarction reduced leukocyte infiltration and extracellular matrix turnover and preserved cardiac function [4].

Etanercept is a soluble TNF-α binding protein with a long half-life. It directly binds to TNF-α reducing the biological effectiveness of TNF-α [5], [6]. Etanercept is frequently used to treat autoimmune disease like rheumatoid arthritis [7], ankylosing spondylitis [8], psoriasis and psoriatic arthritis [9] by acting as a TNF-α inhibitor. It has also been used as a safe drug in patients having psoriasis along with HCV infection [10]. The long-term safety of etanercept in children is well established [11]. However, the effect of etanercept on myocardial ischemia/reperfusion injury is not well understood. In present study, therefore, we investigated the effect of etanercept as an anti-TNF-α therapy on myocardial ischemia/reperfusion rat model and its underlying mechanisms.

## Materials and Methods

### Reagents

Etanercept was purchased from Sigma Chemical Co. (St. Louis, MO, USA). MPO assay kit, CK-MB, cardiac troponin I assay kit and LDH assay kit were purchased from Jiancheng Bioengineering Institute (Nanjing, China). TNF-α ELISA kit was purchased from R&D Corporation, (USA). BCA protein quantification kit was purchased from Bio-Rad (USA). GSH-PX and SOD activity assay kit and MDA content assay kit were purchased from Jiancheng Bioengineering Institute (Nanjing, China).

### Animals

Thirty adult male Sprague-Dawley rats (250–300 g) were purchased from the Center of Experimental Animal in Shandong University, China. All animals used in this study were cared for in accordance with the Guide for the Care and Use of Laboratory Animals published by the United States National Institute of Health (NIH publication no. 85-23, revised 1996), and all procedures were approved by the Committee of Experimental Animals of Shandong University.

### Myocardial ischemia-reperfusion model and experimental protocol

Male Sprague-Dawley rats (250–300 g) were anesthetized i.p. with sodium pentobarbital (Sigma, St. Louis, USA, 40 mg/kg). An intratracheal cannula was inserted and the animals were placed in intermittent positive pressure ventilation with room air. Myocardial ischemia was produced by exteriorizing the heart with a left thoracic incision followed by a slipknot (5–0 silk) around the left anterior descending coronary artery (LAD). After 30 min of ischemia, the slipknot was released and the animal received 120 min of reperfusion. Rats were randomly assigned to three experimental groups. There were 10 rats in each group: (1) sham group: silk was drilled underneath the LAD but the LAD was not ligated; (2) MI/R group: LAD was ligated for 30 min and then allowed 120 min reperfusion with receiving vehicle (0.9% NaCl i.v.); (3) MI/R + Etanercept group: Etanercept (10 mg/kg, i.v.) was administered 10 min before reperfusion.

### Detection of cardiac function

Rats were anesthetized i.p. with sodium pentobarbital (Sigma, St. Louis, USA, 40 mg/kg), and a catheter was inserted into the left ventricle through the right common carotid artery for measurement of left ventricular function, which included left ventricular ejection fraction (LVEF), left ventricular end-diastolic pressure (LVEDP) and the maximal rate of rise and decline of ventricular pressure (±dp/dt[max]). The cardiac function was recorded and stored with the AcqKnowledge 3.8.1 software package and a Biopac Data Acquisition System (Biopac Systems Inc., USA).

### Assay of myocardial infarct area

After reperfusion, myocardial infarct size was determined by means of a double-staining technique and a digital imaging system (infarct area/area at risk×100%) [12]. After reperfusion, coronary blood flow was again blocked and Evans blue (2%, 4 ml) was injected by the rapid distribution of the right ventricle into the body. The heart was quickly removed to a −20°C refrigerator for cryopreservation. The heart was cut into 1 mm slices, placed in 1% 2,3,5-triphenyltetrazolium chloride (TTC) solution, incubated for 15 min, and then placed in 4% formaldehyde solution overnight. Evans blue stained area (blue staining, non-ischemic area), TTC stained area (red staining, ischemic area) and non-TTC stained area (white, infarct area) were analyzed with a digital imaging system by computer. Myocardial infarct area (infarct area/area at risk%, INF/AAR%) was calculated.

### Determination of cardiomyocyte apoptosis

At the end of reperfusion, myocardial apoptosis was analyzed by terminal deoxynu-cleotidyl transferase dUTP nick end labeling assay (TUNEL) using an in situ cell death detection kit. A double-staining technique was used, in brief, TUNEL staining for apoptotic cell nuclei and 4′,6-diamino-2-phenylindole (DAPI) staining for all myocardial cell nuclei as described previously [13]. The index of apoptosis was expressed by the number of positively stained apoptotic cardiomyocytes/the total number of cardiomyocytes counted×100%.

### Quantitative real-time PCR for TLR4

Quantitative real-time PCR analyses of TLR4 expression were performed using RNAs isolated from experimental and control samples. For quantitative real-time PCR, total RNAs were purified from hearts using Trizol reagent according to the manufacturer’s instructions. RNA was transcribed to cDNA with PrimeScript RT reagent Kit. Then qRT-PCR was performed using the CFX96™ real-time system, and the relative gene expression was normalized to internal control gapdh. The analysis of the melting curve of each amplified PCR product and the visualization of the PCR amplicons on 1.5% agarose gels allowed to control the specificity of the amplification. Primer sequences for SYBR Green probes of target genes are as follows:

TLR4-f: GGCATCATCTTCATTGTCCTTG TLR4-r: AGCATTGTCCTCCCACTCG Act-f: GGAGATTACTGCCCTGGCTCCTA Act-r: GACTCATCGTACTCCTGCTTGCTG.

### Immunohistochemical analysis of TLR4 expression

Before immunohistochemical examination, 3-µm slices from pretreated myocardium tissue were placed in a bathing solution of 3% H_2_O_2_ and 60% methanol PBS for 30 min and then treated with 0.01 mol/L sodium citrate buffer at 95°C in a microwave oven for 13 min (antigen retrieval). Thereafter, specimens were treated with 5% normal goat serum and 5% bovine serum albumin in PBS. Before each step, sections were rinsed three times in PBS buffer. Incubation with primary Anti-TLR4 antibodies was performed in a PBS-based solution of 1% bovine serum albumin for 12 h at 4°C in the recommended dilutions. After rinsing with PBS, sections were incubated with the corresponding secondary biotinylated goat anti-rabbit antibodies Envison+ for 1 h at room temperature. A streptavidin/horseradish peroxidase complex was then applied as a detection system (1∶100 dilution) for 1 h. Finally, staining was developed with 3,3′-diaminobenzidine tetra-hydrochloride in 0.05 mol/L Tris–HCl buffer and 0.1% H_2_O_2_. Negative control sections were incubated without the primary antibody. All dates in this study were analyzed by software ‘‘Image Pro Plus’’ (Media Cybernetics Corporation, DC).

### Western blotting analysis of NF-κB expression

At the end of reperfusion, rats’ myocardial tissues were harvested and washed with ice-cold normal saline and homogenized on ice in phosphate buffered saline. After centrifuged at 3000 rpm for 5 min, the supernatant was discarded. The homogenate was incubated in lysis buffer including protease inhibitor, PMSF, etc. for 20 min with vortex-mixing at 4°C, filtered and centrifuged at 12,000 g for 20 min. The resulting supernatants were the proteins, of which the concentrations were determined by Bradford's method, with bovine serum albumin serving as a calibrator. The immunoblots were probed with anti-NF-κB antibodies overnight at 4°C followed by incubation with the corresponding secondary antibodies at room temperature for 1 h. The blots were visualized with ECL-Plus reagent.

### Detection of TNF-α Level

After reperfusion, the levels of TNF-α in myocardial tissue homogenate and serum were detected in strict accordance with manufacturer’s instructions. BCA kit was used to detect the protein quantization.

### Determination of myeloperoxidase (MPO) level

After reperfusion, the myocardial tissue was placed at −70°C for preservation. MPO test kit was used to detect level of MPO in the myocardial tissue according to manufacturer’s instruction.

### Detection of creatine kinase-MB (CK-MB) and cardiac troponin I activity

After reperfusion, blood was taken from the carotid artery and was placed at room temperature for 30 min. Then, the serum was collected by centrifugation and placed at −70°C for preservation. According to manufacturer’s instruction, the CK-MB and cardiac troponin I assay kit were utilized to detect the serum CK-MB and cardiac troponin I activity.

### Determination of lactate dehydrogenase (LDH) level

After reperfusion, blood was taken from the carotid artery and was placed at room temperature for 30 min. Then, the serum was collected by centrifugation and placed at −70°C for preservation. The extent of cell injury was monitored by measuring LDH leakage. According to the manufacturer’s instruction, the CK test kit was utilized to detect the serum LDH level.

### Detection of SOD, GSH-PX activity and MDA content

After reperfusion, myocardial tissue was homogenized in ice cold phosphate buffer to make a 10% homogenate. Then the homogenate was centrifuged at 3000 rpm for 15 min. Superoxide dismutase (SOD), glutathione peroxidase (GSH-PX), and malondialdehyde (MDA) in the supernatant were measured by using commercially available kits. All assays were conducted according to the manufacturer’s instructions.

### Statistical analysis

Data is presented as means ± S.D. The significance of differences among groups was evaluated by a Student’s t-test for unpaired data or Dunnett’s t-test for multiple comparisons preceded by one-way analysis of variance (ANOVA). For all test, *P*<0.05 was considered as statistically significant.

## Results

### Etanercept ameliorated cardiac dysfunction induced by MI/R

**Figure 1:**
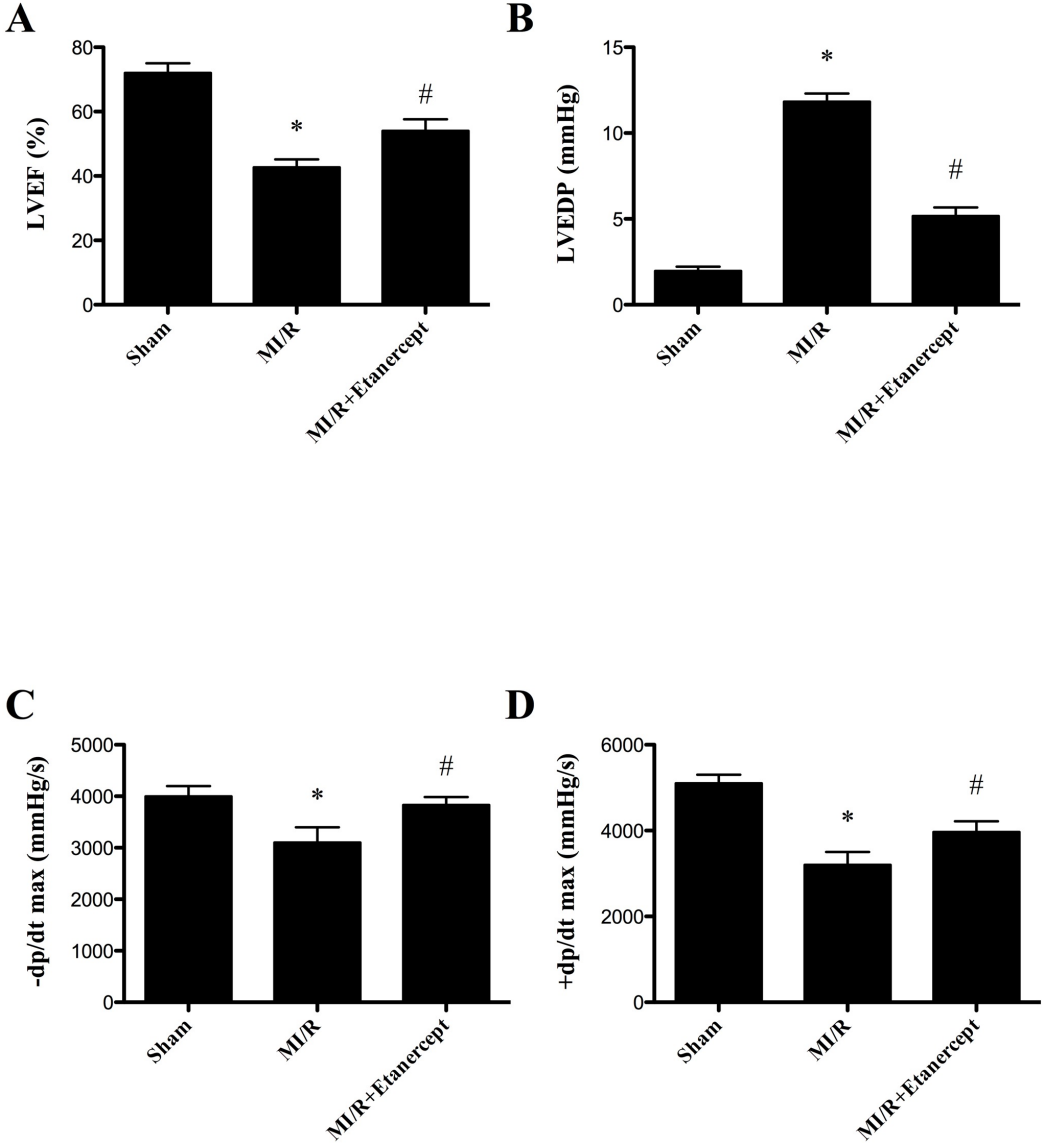
Effect of etanercept on cardiac function. (A) The effect of etanercept on LVEF. (B) The effect of etanercept on LVEDP. (C) The effect of etanercept on –dp/dx max. (D) The effect of etanercept on +dp/dx max. LVEF, left ventricle ejection fraction; LVEDP, left ventricle end-diastolic pressure. Data were expressed as mean ± SD (n = 10 in each group). *P<0.01 versus the sham group, ^#^ *P*<0.05 versus MI/R group.

Compared with the MI/R group, etanercept significantly elevated LVEF, +dp/dt max and –dp/dt max and decreased LVEDP (*P*<0.05) (Figure 1).

### Etanercept reduced the myocardial infarction area induced by MI/R

**Figure 2:**
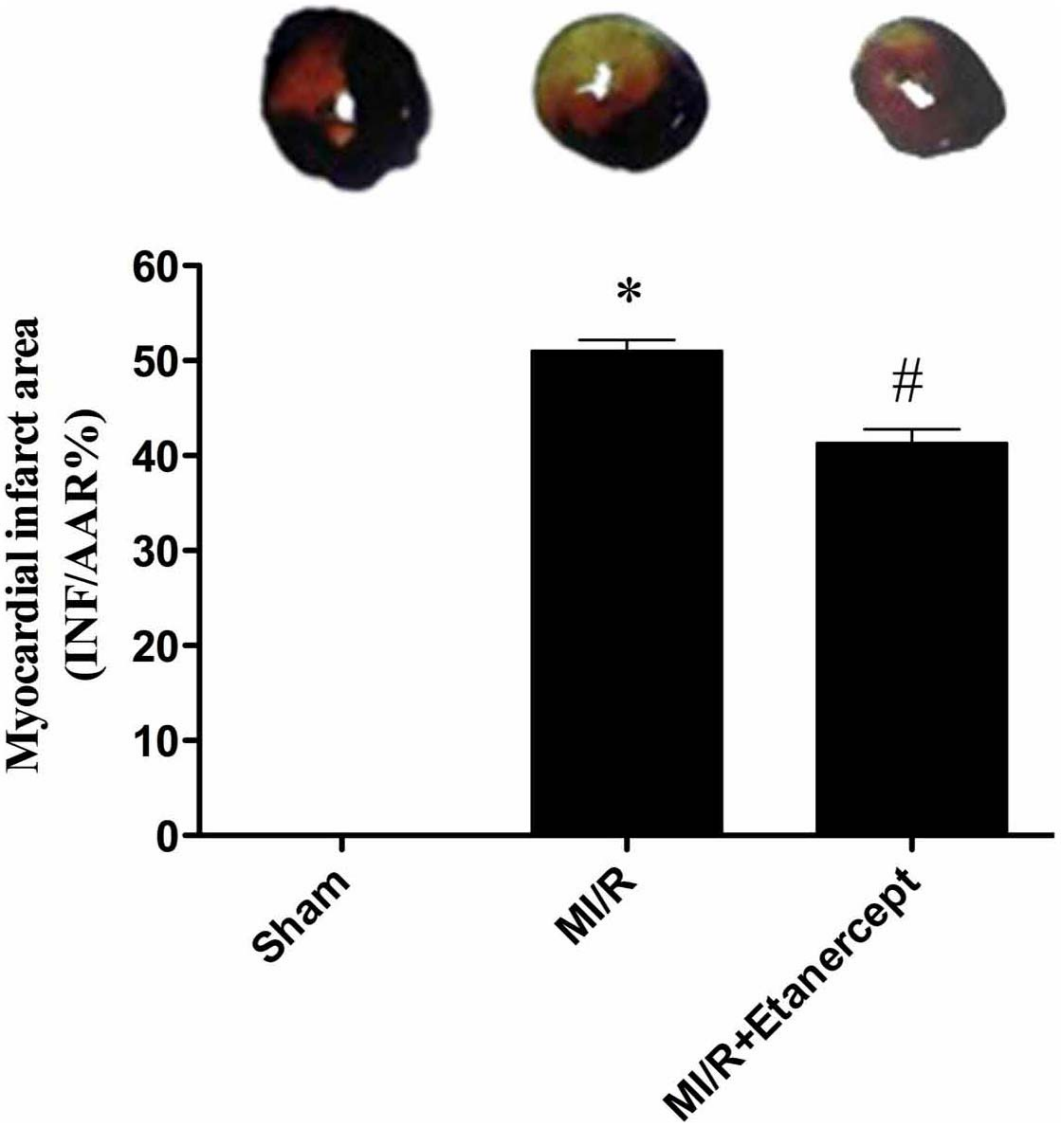
Effect of etanercept on the myocardial infarction area induced by MI/R. Data were expressed as mean ± SD (n = 10 in each group). *P<0.01 versus the sham group, ^#^ *P*<0.05 versus MI/R group.

MI/R induced a significant infarction area. Compared with the MI/R group, etanercept reduced myocardial infarction area significantly (*P*<0.05) (Figure 2).

### Etanercept reduced cardiomyocyte apoptosis induced by MI/R

**Figure 3:**
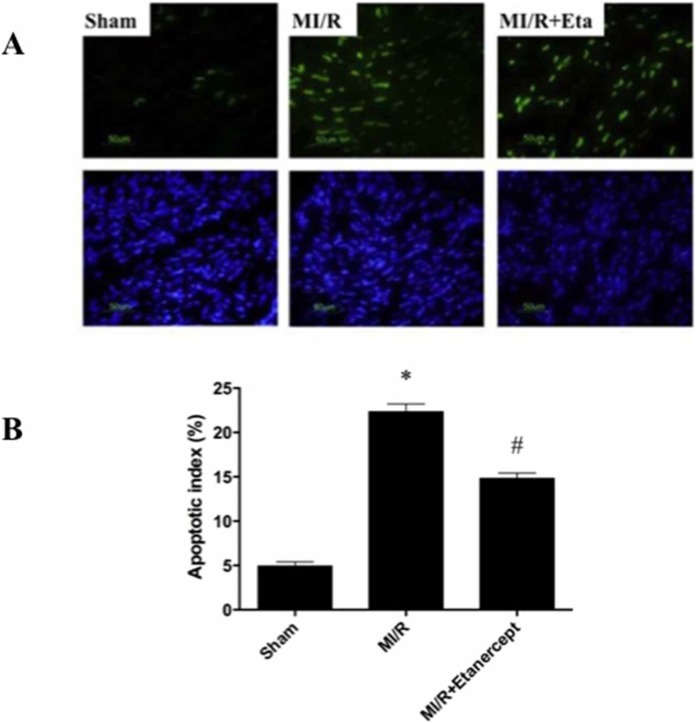
Effect of etanercept on cardiomyocyte apoptosis induced by MI/R. (A) TUNEL staining. (B) Quantitative analysis of percentage of cardiomyocyte apoptosis. Data were expressed as mean ± SD (n = 10 in each group). *P<0.05 versus the sham group, ^#^ *P*<0.05 versus MI/R group.

In myocardial tissue from the sham group, a very low level of TUNEL-positive staining was detected. A significant number of TUNEL-positive cells were observed in myocardial tissue from hearts subjected to MI/R. Administration of etanercept at 10 min before reperfusion exerted a significant anti-apoptotic effect as evidenced by reduced TUNEL-positive staining (Figure 3).

### Etanercept down-regulated TLR4 gene and protein expressions in the ischemia area in the heart subjected to MI/R

**Figure 4:**
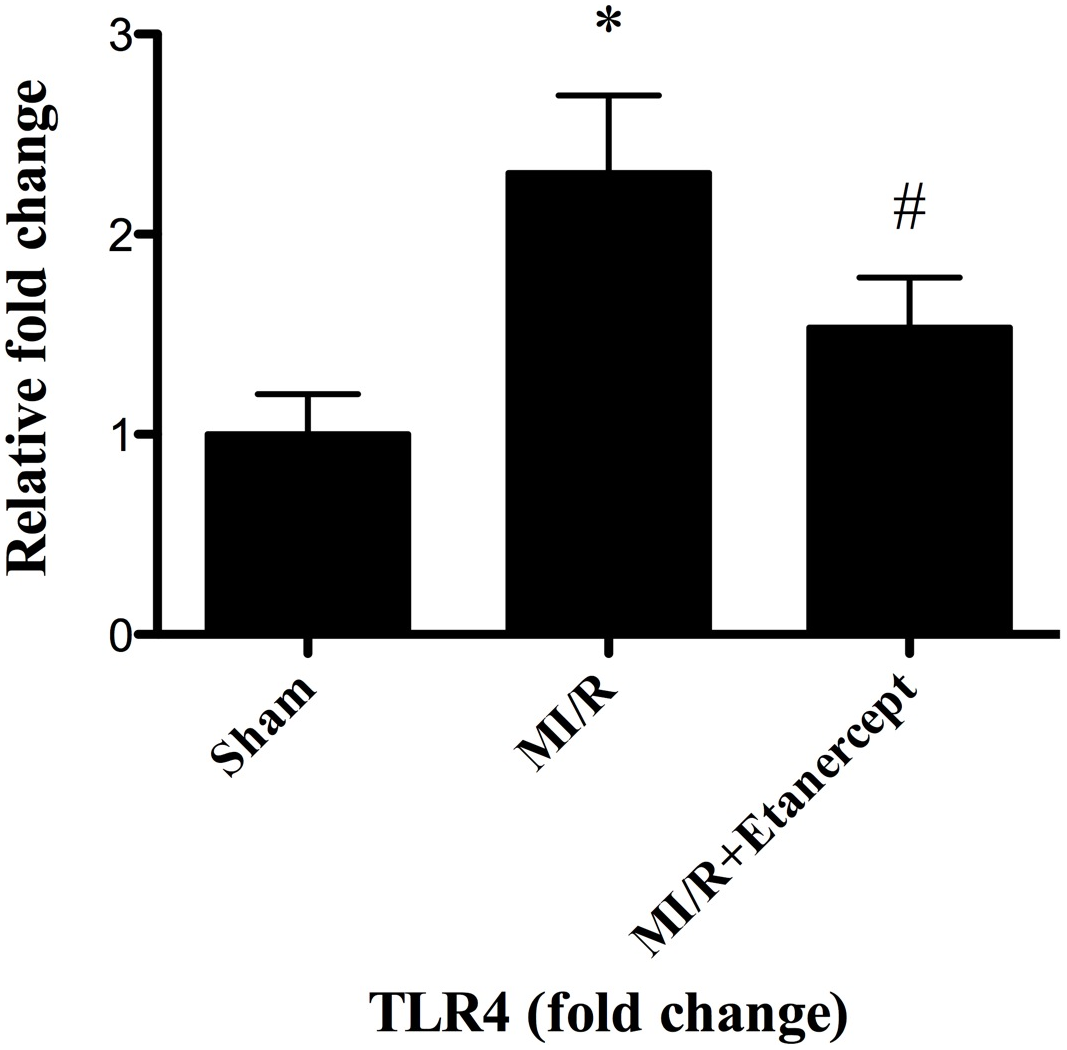
Effect of etanercept on TLR4 gene expression in the ischemia area in the heart subjected to MI/R. Data were expressed as mean ± SD (n = 10 in each group). *P<0.05 versus the sham group, ^#^ *P*<0.05 versus MI/R group.

The results from real-time PCR suggested that the expression of TLR4 gene was up-regulated in the ischemia area in MI/R group. Administration of etanercept significantly decreased the TLR4 gene expression (Figure 4).

**Figure 5:**
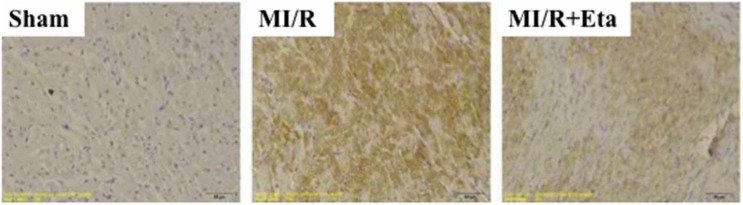
Immunohistochemical analysis of myocardial TLR4 expression. TLR4 is brown staining. Sham, sham group; MI/R, MI/R group; MI/R+Eta, MI/R+ etanercept group. Data were expressed as mean ± SD (n = 10 in each group).*P<0.05 versus the sham group, ^#^ *P*<0.05 versus MI/R group.

In the MI/R group, the localization of TLR4 in DAB staining showed that the expression of TLR4 was highly elevated. With the administration of etanercept, the expression of TLR4 was significantly reduced both in the ischemia area (Figure 5).

Etanercept down-regulated NF-κB expression in the ischemia area in the heart subjected to MI/R.

**Figure 6:**
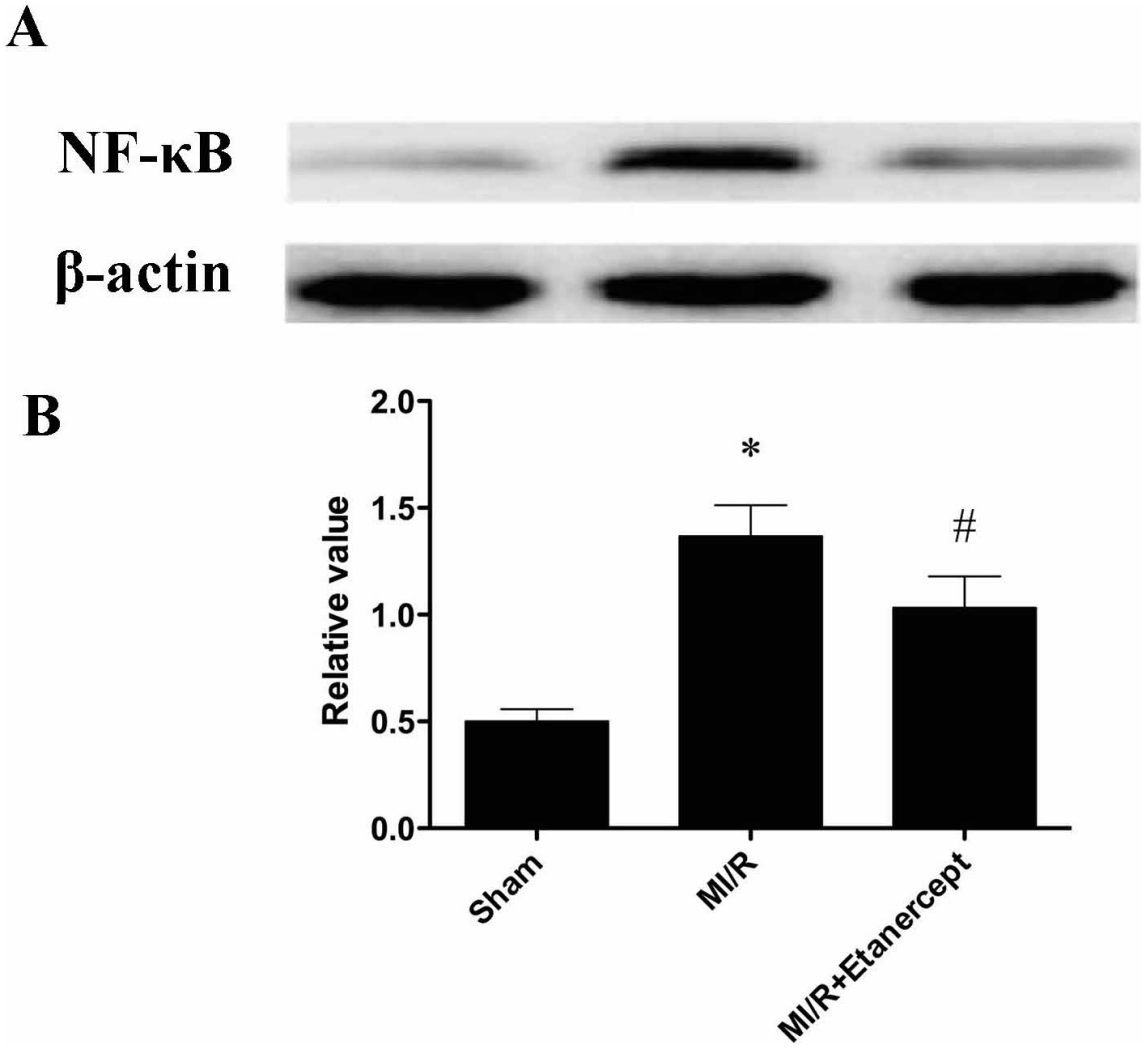
Effect of etanercept on NF-κB expression in the ischemia area in the heart subjected to MI/R. (A) Representative immunoblots of samples from rat ventricles subjected to different treatment groups. (B) Quantitative densitometric analysis of NF-κB protein with β-actin as an internal standard. Data were expressed as mean ± SD (n = 10 in each group). *P<0.05 versus the sham group, ^#^ *P*<0.05 versus MI/R group.

To further study the effect of etanercept on TLR4 mediated signaling, NF-κB, a downstream molecule of TLR4 was determined. The results from Western blot showed that the expression of NF-κB was up-regulated in the ischemia area in MI/R group. Administration of etanercept significantly decreased NF-κB expression (Figure 6).

### Etanercept reduced TNF-α levels in serum and MI/R tissue

**Figure 7:**
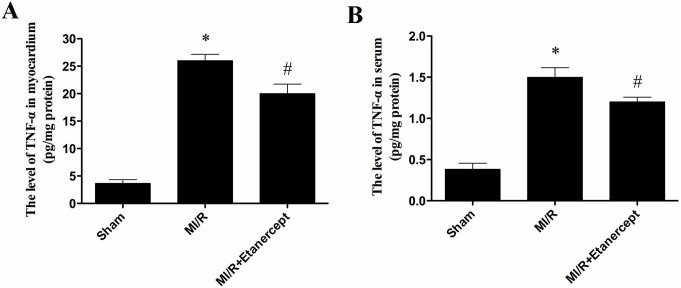
The comparison of levels of TNF-α in myocardium and serum in each group. Compared with the MI/R group, etanercept reduced TNF-α level significantly. Data were expressed as mean ± SD (n = 10 in each group). *P<0.05 versus the sham group, ^#^ *P*<0.05 versus MI/R group.

The MI/R injury results in production of large amount of TNF-α. Thus, myocardial and serum TNF-α levels were examined. In Figure 7, compared with the MI/R group, Etanercept significantly decreased the levels of TNF-α in both myocardium and serum (*P*<0.05) (Figure 7).

### Etanercept inhibited neutrophil infiltration in MI/R tissue

**Figure 8:**
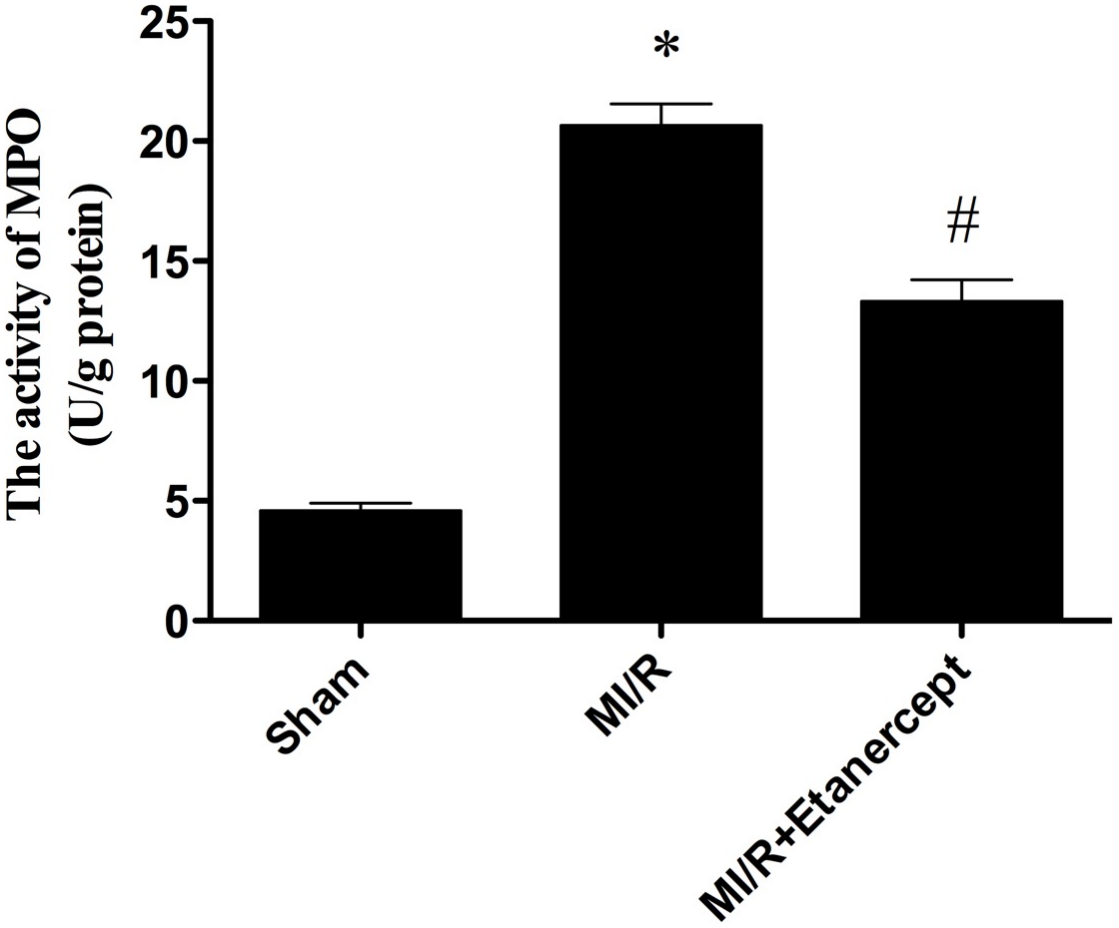
The comparison of MPO activity in each group. The MPO activity in the sham group was relatively lower, while the MPO activity in MI/R group was significantly increased, which was blocked by etanercept. Data were expressed as mean ± SD (n = 10 in each group). *P<0.05 versus the sham group, ^#^ *P*<0.05 versus MI/R group.

Neutrophil contains a certain amount of myeloperoxidase (MPO), accounting for 5% of dry cell weight. So the activity of MPO in the myocardium can be considered as the indication of neutrophil infiltration. As shown in Figure 8, the MPO activity in the sham group was relatively lower, while the MPO activity in MI/R group was significantly increased (*P*<0.05). Etanercept significantly decreased myocardial MPO activity (*P*<0.05) (Figure 8).

### Etanercept reduced the activity of serum CK-MB and cardiac troponin I in MI/R rats

**Figure 9:**
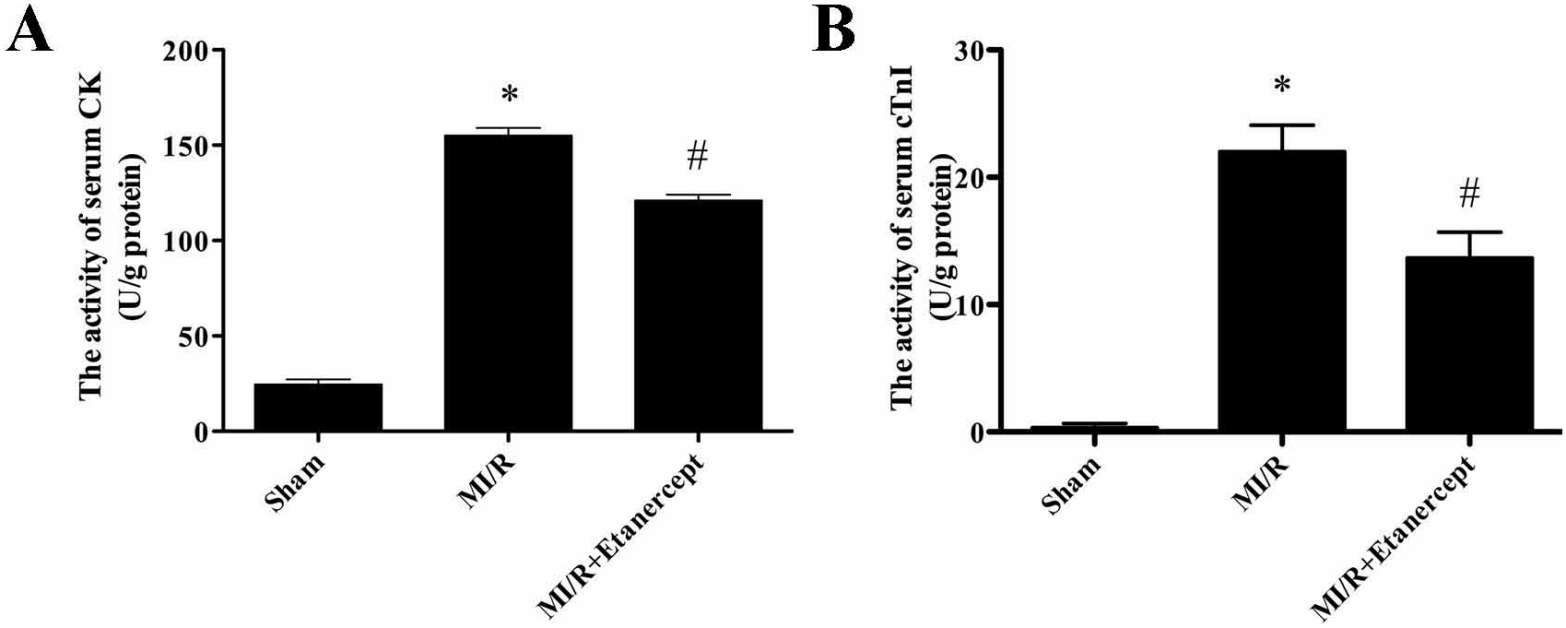
The comparison of CK and cTnI activity in each group. The CK and cTnI activity in the sham group was relatively lower, while the CK and cTnI activity in MI/R group was significantly increased, which was inhibited by etanercept. Data were expressed as mean ± SD (n = 10 in each group). *P<0.05 versus the sham group, ^#^ *P*<0.05 versus MI/R group.

As shown in Figure 9, the activity of CK-MB and cardiac troponin increased significantly in the MI/R group compared with the sham group (*P*<0.05). CK-MB and cardiac troponin I activity decreased significantly in the MI/R+Etanercept group compared with the MI/R group (*P*<0.05) (Figure 9).

### Etanercept reduced LDH Level in MI/R rats

**Figure 10:**
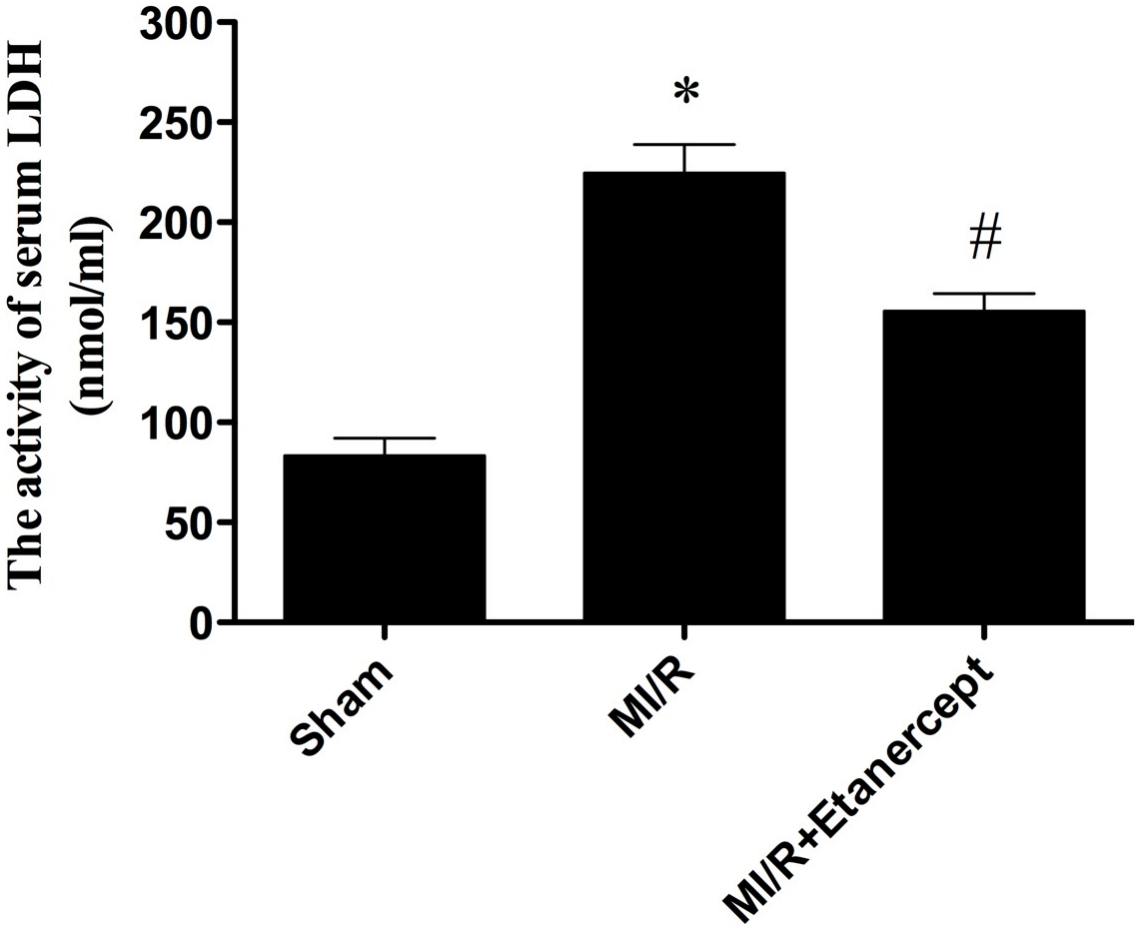
The comparison of LDH activity in each group. The LDH activity in the sham group was relatively lower, while the LDH activity in MI/R group was significantly increased, which was inhibited by etanercept. Data were expressed as mean ± SD (n = 10 in each group). *P<0.05 versus the sham group, ^#^ *P*<0.05 versus MI/R group.

As shown in Figure 10, the activity of LDH increased significantly in the MI/R group compared with the sham group (*P*<0.05). LDH activity decreased significantly in the MI/R+Etanercept group compared with the MI/R group (*P*<0.05) (Figure 10).

### Etanercept elevated antioxidant enzymes activities and decreased MDA content

**Figure 11:**
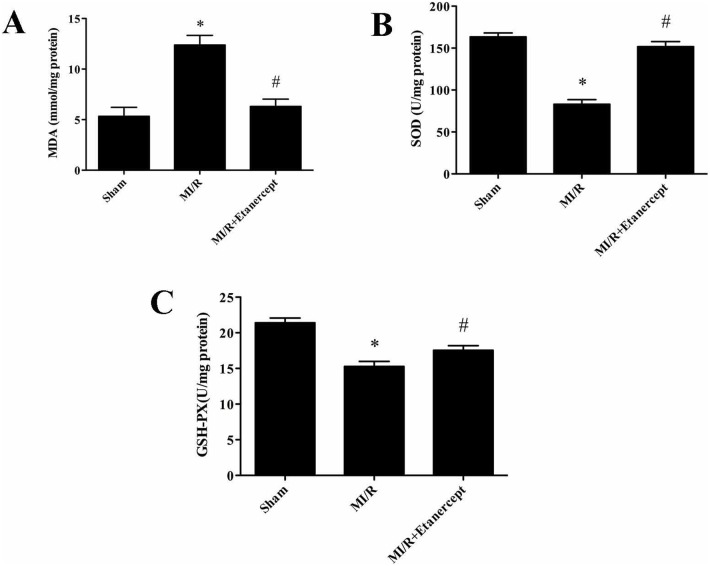
Etanercept ameliorates oxidative stress after myocardial ischemia/reperfusion injury. Etanercept significantly decreased (A) the content of malondialdehyde (MDA) and (B) enhanced superoxide dismutase (SOD); and (C) glutathione peroxidase (GSH-PX) activities in ischemia reperfusion rats’ heart. Data were expressed as mean ± SD (n = 10 in each group). *P<0.05 versus the sham group, ^#^ *P*<0.05 versus MI/R group.

To investigate whether Etanercept affects oxidative stress damage, we evaluated the activities of antioxidant enzyme and the content of MDA. The activities of GSH-PX and SOD were decreased significantly in the MI/R group as compared with the sham group (*P*<0.05). However, Etanercept treatment induced significant elevation of GSH-PX and SOD activities compared with the MI/R group (*P*<0.05). The content of MDA is an index of lipid peroxidation, which increased significantly after myocardial I/R injury (*P*<0.05). Etanercept treatment significantly decreased the MDA content compared with the MI/R group (*P*<0.05) (Figure 11).

## Discussion

The major findings in the present study are: (1) Etanercept protects heart from ischemia/reperfusion injury by inhibiting apoptosis. (2) Etanercept attenuates inflammatory reaction induced by I/R injury by inhibiting TLR4/NF-κB signaling. (3) Etanercept attenuates inflammatory reaction induced by I/R injury through inhibiting neutrophil infiltration and TNF-α production. (4) Etanercept attenuates MI/R injury by elevating SOD and GSH-PX activity and decreasing MDA content.

Inflammatory reaction plays an important role in myocardial ischemia/reperfusion injury [1]. The release of inflammatory cytokines and the aggregation and infiltration of inflammatory cells are the key steps in inflammation [14].

TNF-α is secreted mainly by macrophages, which is likely to promote inflammatory cascade by increasing releases of other proinflammatory cytokines and influencing neutrophil recruitment [15]. TNF-α, as an important cytokine in inflammation, plays an important role of initiation in the inflammation induced by MI/R [16]. TNF-α can induce the release of other inflammatory mediators, increase the expression of cell adhesion factor, and promote neutrophil adhesion to endothelial cells. In addition, TNF-α has a negative inotropic effect, which can inhibit myocardial contractility, and lower blood pressure. TNF-α can also induce cardiomyocyte apoptosis and participate in ventricular remodeling [17]. Previous studies suggest that the level of TNF-α increases significantly after MI/R [18], while the administration of TNF-α monoclonal antibody attenuated edema, and is conducive to cardiac function recovery [19].

MI/R injury seems to be induced in part by neutrophil activation. The underlying mechanisms include: (1) Cell damage caused by the release of oxygen free radicals, proteolytic enzymes, and cytotoxic substances. (2) The released inflammatory mediators cause vascular endothelial cell damage, increased vascular permeability, and edema. (3) Further activation of inflammatory cells increase further the inflammatory response [20]. (4) Neutrophil adhesion to vascular endothelium and small blood vessels occlusion result in no-reflow phenomenon.

Previous studies have demonstrated the link between neutrophil and ischemia/reperfusion injury. Removal of neutrophils or drug inhibition of neutrophil activity has been shown to reduce ischemia/reperfusion injury [21], [22]. In the present study, we found that neutrophil accumulation and TNF-α production in the MI/R group increased significantly. And etanercept reduces neutrophil accumulation and TNF-α production, indicating that etanercept inhibits neutrophil accumulation and TNF-α production and therefore it attenuated neutrophil-mediated ischemia/reperfusion injury.

In human, it is suggested that etanercept treatment dramatically increased serum TNF-α level [23]. However, in this study, we found that etanercept treatment dramatically decreased serum TNF-α level in rats. On one hand, our study is an animal study. It is probably that human and rats react differently to etanercept treatment. On the other hand, even if human and rats react the same to etanercept treatment, there are still many factors leading to the different levels in serum TNF-α level. Maybe the infarct area is one of factors. When the infarct area reaches a certain level (50% or even more), etanercept treatment may be a beneficial factor, leading to a decrease in serum TNF-α level to some extent. However, when the infarct area is at a relatively low level, etanercept treatment may be a detrimental factor, leading to the increase in serum TNF-α level. In medicine, there is no absolute conclusion, and only in certain conditions, can the so-called conclusion be right. Of course, this is the limitation in our study and the human study published in Heart. And further study is needed to find out the exact causes.

Under physiological conditions ROS are generated at low levels and play important roles in signaling and metabolic pathways [24], however, under pathologic conditions such as MI/R, their overproduction leads to oxidative stress, causing cell damage to nervous tissue, which may lead to DNA oxidation, promoting chain reactions of membrane lipid peroxidation, and alterations in membrane fluidity [25], [26]. ROS produces malondialdehyde (MDA), an end product of lipid peroxidation. Therefore in the present study the level of MDA was estimated to estimate extent of ROS. Our result showed that the elevated level of MDA was markedly decreased by treatment with etanercept, indicating that the cardioprotection conferred by etanercept may be attributed to attenuating lipid peroxidation following myocardial ischemia/reperfusion. The overproduction of ROS can be detoxified by endogenous antioxidants, causing their cellular stores to be depleted [27]. Superoxide dismutase (SOD) and glutathione peroxidase (GSH-PX) are thought to be two dominant enzymes acting as free radical scavengers that could prevent ROS generation [28]. SOD scavenges the superoxide anion radical (O^−^_2_) by catalyzing its dismutation to H_2_O_2_, which is scavenged to water by GSH-PX at the expense of glutathione [29]. In the present study, etanercept was suggested to be effective in stimulating the activities of SOD and GSH-PX. Our data suggests that etanercept protects myocardial I/R injury through the amelioration of oxidative stress.

In conclusion, the present study demonstrates that etanercept attenuates myocardial ischemia/reperfusion injury. And the protective effect of etanercept is closely associated with the inhibition of apoptosis, neutrophil infiltration and TNF-α production, the increase of SOD and GSH-PX activity and decrease of MDA content.
